# Supplementary material for: Clinical relevance for circulating cold-inducible RNA-binding protein (CIRP) in patients with adult-onset Still’s disease
Source: PLoS One. 2021 Aug 5;16(8):e0255493. doi: 10.1371/journal.pone.0255493 (PMC8341607; doi:10.1371/journal.pone.0255493)
Supplement: S1 Dataset — (DOCX) [file pone.0255493.s001.docx]

**What data are required and what is meant by minimal data set? PLOS defines the “minimal data set” to consist of the data set used to reach the conclusions drawn in the manuscript with related metadata and methods, and any additional data required to replicate the reported study findings in their entirety. Authors do not need to submit their entire data set, or the raw data collected during an investigation. Please submit the following data.**

**・The values behind the means, standard deviations and other measures reported;**

**・The values used to build graphs;**

**・The points extracted from images for analysis.**

| **Fig. #** | **Median** | **IQR** | **Statistical method used** | **P value** | **# sample** |
| --- | --- | --- | --- | --- | --- |
| **Fig.1** |  |  | Kruskal-Wallis test  Games-Howell test was used for Post hoc pairwise analysis. |  |  |
| HC | 2.8 | 1.4-4.9 |  | p < 0.001* | 15 |
| AOSD | 9.6 | 6.1-13.7 |  |  | 44 |
| RA | 3.2 | 1.9-3.8 |  | p < 0.001* | 50 |
| SLE | 2.2 | 1.9-2.4 |  | p < 0.001* | 20 |
| **Fig. 2** |  |  | Mann-Whitney U test |  |  |
| Active AOSD | 11.6 | 7.4-17.8 |  | p = 0.007 | 27 |
| Inactive AOSD | 6.2 | 4.2-11.5 |  |  | 17 |
| **Fig.3** |  |  | Spearman’s rank correlation  coefficient |  |  |
| CIRP | 9.6 | 5.8-14.0 |  |  | 44 |
| Ferritin | 1015.5 | 298.0-3896.0 |  | p = 0.002** | 44 |
| CRP | 5.5 | 2.8-10.4 |  | p = 0.044** | 44 |
| Disease Activity | 3.0 | 2.0-4.8 |  | p = 0.002** | 44 |
| IL-18 | 40247.7 | 10220.0-129564.1 |  | p = 0.038** | 43 |
| **Fig.4** |  |  | Kruskal-Wallis test |  |  |
| Polycyclic systemic type | 10.2 | 6.2-17.8 |  | p = 0.484 | 23 |
| Monocyclic systemic type | 9.9 | 5.2-14.8 |  |  | 15 |
| Chronic arthritis type | 6.8 | 5.1-10.6 |  |  | 6 |
| **Fig.5** |  |  | Wilcoxon signed-rank test |  |  |
| CIRP before | 7.4 | 6.2-13.5 |  | p < 0.001 | 8 |
| CIRP after | 2.4 | 2.2-3.0 |  |  | 8 |
| Ferritin before | 1502 | 872-2764 |  | p < 0.001 | 8 |
| Ferritin after | 46 | 41-53 |  |  | 8 |
| Pouchot’s score before | 3 | 3-5 |  | p < 0.001 | 8 |
| Pouchot’s score after | 0 | 0-1 |  |  | 8 |

* Compared with AOSD patients.

** Compared with CIRP
