# Supplementary material for: Clinical relevance for circulating cold-inducible RNA-binding protein (CIRP) in patients with adult-onset Still’s disease
Source: PLoS One. 2021 Aug 5;16(8):e0255493. doi: 10.1371/journal.pone.0255493 (PMC8341607; doi:10.1371/journal.pone.0255493)
Supplement: Attachment — Submitted filename: Response to Reviewers.docx [file pone.0255493.s002.docx]

Response to Reviewer #1

Comment

The authors answered all questions well. In my opinion, I do not think I can give some suggestions for current version.

Response

I am grateful for your comment

Response to Reviewer 2

We wish to express our appreciation to your insightful comments on our paper. The comments have helped us significantly improve the paper.

Comment

The authors added SLE and RA as disease control, but the medication seems diverse in RA patients (the medication ofSLE patients not described). The influence of medication such as glucocorticoids may also affect the level of CIRP.

Response

I appreciate for your critical comment. All SLE patients was untreated. Among 50 RA patients, 8 RA patients was untreated. We added the comparison between untreated AOSD and untreated RA as follows, “Serum levels of AOSD patients were significantly higher than that of untreated RA (2.6 ng/mL, [IQR; 1.7-3.8], p<0.001).”
